# Externalising moods and psychological states in a cloud based system to enhance a pet-robot and child’s interaction

**DOI:** 10.1186/s12938-016-0180-3

**Published:** 2016-07-15

**Authors:** Ferran Larriba, Cristóbal Raya, Cecilio Angulo, Jordi Albo-Canals, Marta Díaz, Roger Boldú

**Affiliations:** 1 Automatic Control Department, Universitat Politècnica de Catalunya, Pau Gargallo 5, 08028 Barcelona, Spain; 2 La Salle BCN, Universitat Ramon Llull, Quatre Camins, 08022 Barcelona, Spain; 3 CEEO, Tufts University, 200 Boston Ave, Medford, MA 02155 USA; 4 Research Centre for Dependency Care and Autonomous Living, Universitat Politècnica de Catalunya, Rambla Exposició 59-69, 08800 Vilanova I La Geltrú, Spain; 5 Fluid Interfaces Group, MIT Media Laboratory, 77 Massachusetts Ave, Cambridge, MA 02139 USA

## Abstract

**Background:**

This PATRICIA research project is about using pet robots to reduce pain and anxiety in hospitalized children. The study began 2 years ago and it is believed that the advances made in this project are significant. Patients, parents, nurses, psychologists, and engineers have *adopted* the Pleo robot, a baby dinosaur robotic pet, which works in different ways to assist children during hospitalization.

**Methods:**

Focus is spent on creating a wireless communication system with the Pleo in order to help the coordinator, who conducts therapy with the child, monitor, understand, and control Pleo’s behavior at any moment. This article reports how this technological function is being developed and tested.

**Results:**

Wireless communication between the Pleo and an Android device is achieved. The developed Android app allows the user to obtain any state of the robot without stopping its interaction with the patient. Moreover, information is sent to a cloud, so that robot moods, states and interactions can be shared among different robots.

**Conclusions:**

Pleo attachment was successful for more than 1 month, working with children in therapy, which makes the investment capable of positive therapeutic possibilities. This technical improvement in the Pleo addresses two key issues in social robotics: needing an enhanced response to maintain the attention and engagement of the child, and using the system as a platform to collect the states of the child’s progress for clinical purposes.

## Background

Therapeutic robots that work to diagnose, rehabilitate, and assist in surgery, are all designed to motivate and assist patients with psychological conditions. Robo-therapy consists of the interaction between patients and robotic creatures in order to help patients build a positive attitude towards fighting a disease [1].

Since pet robots are mainly considered in this study, focus is spent on robots that can be categorized with similar therapeutic effects as in animal-assisted therapy [2]. Unfortunately with animal-assisted therapy, these animals are not readily available. Concerns towards dog bites, allergies, or diseases have led to high restriction or removal of this type of therapy in hospitals and nursing homes. Other issues include the need of a trained professional at all times around these animals, and sessions that are restricted to a schedule. Robotic therapy has emerged to combat these issues.

The Program *Child Life* was started in 2004 at Hospital Sant Joan de Déu (HSJD), in Barcelona, Spain, with the purpose of designing pioneering techniques to improve a child’s experience by reducing pain and anxiety during hospitalization [3]. In 2010, more than 200 children and teenagers along with their families have participated in this program.

The PATRICIA project [4] is based on the use of social robots with the same goal. HSJD has teamed up with the Universitat Politècnica de Catalunya (UPC) and La Salle Universitat Ramon Llull (La Salle URL) to collaborate as technological partners. This is an innovative interdisciplinary project addressed to improve the quality of life of hospitalized children that may face many challenges. Therapeutic robots act as a robotic companion that can stand alongside a person coping with a psychologically demanding situation. The work behind this therapy is not an easy endeavor. Companies such as Fujitsu, Innvo Labs, and PARO Robots have been working on this for decades and it is only in the last 5 years that they have gained studies with positive results.

A huggable Teddy Bear is being developed by Fujitsu as a therapeutic companion for hospitals and nursing homes to be used in health care, education, and social communication applications [6]. It uses a dozen sensors to recognize facial expressions and movements from a patient with a camera located on its nose. It is intended to record the patient’s emotional state and react accordingly using a range of 300 shared actions.

PARO is a baby seal shaped robot designed by Takanori Shibata in Japan in 1993, but was not commercialized until 2004 [5]. It is equipped with five kinds of sensors: temperature, touch, light, audio and position sensors. Additionally Paro is able to learn a person’s behavior. This pet offers similar benefits as animal assisted therapy, and is used to treat people with Alzheimer’s and other related disabilities.

ROMIBO is an open source coded therapeutic robot [7] specially designed to the research and treatment of autism in children. It uses wireless communication in order to be remotely controlled.

Finally, the Pleo, our chosen platform, is a robot that imitates a Camarasaurus dinosaur. It exhibits an appealing expressiveness and consists of an array of different behavior and moods. Pleo has been tested in several research studies [4, 8–10] that have been focused on the effect of pet robots in long-term interactions with children. Other interesting research with this robotic platform is robot ethics [11], which plays an important role in robot-therapy.

### Robot autonomy

Four degrees of Pleo’s autonomy can be deployed when interacting with a child:

- *Full autonomous behaviour according to implicit, opaque to users, internal states:* Pleo always acts according to its own criteria. The problem is that in this modality, Pleo’s behaviour is not totally predictable by the user at any time but may be inferred, anticipated or understood by the user according to a previous interaction.
- *Full autonomous behaviour according to observable internal states:* The conductor of the interaction between a patient and Pleo can watch Pleo’s internal states through a graphical interface that externalizes or makes Pleo’s internal states transparent in order to facilitate management of an interaction.
- *External control of Pleo’s states:* The coordinator is allowed to modify or control the robot by changing its internal states and allowing the robotic platform perform its correlative activity.
- *External control of Pleo’s behaviour:* Allows for fully tele-operated control of movements and actions of Pleo. It always requires the presence of the coordinator.

It has been found that the best choice for testing Pleo in a hospital is located in the second and the third levels, thus this will be the approach in this research.

## Methods

Pleo’s platform is capable of helping children at HSJD and their families improve their treatment. This research group presents a new generation of health care robots—combining cloud robotics and artificial intelligence—to provide children with an effective and individualized assistance to their therapy [12].

The goal is to supply each patient with a personal Pleo that uses this cloud multi-agent system to perceive, collect and share the status on a child. Using artificial intelligence, the behaviour of every patient’s robot can be modified. Finally, since this information is in the cloud, the system can explore the most effective actions to carry out to improve its own patient’s experience.

Until now, it is not possible to modify the software system of Pleo since the software is not open sourced. However, it does allow users to modify some values or commit to some precise actions. For example, it is able to notify people about how hungry it is, how happy is it, and do various tasks such as ask a person to go out on a walk, or perform a trick such as giving a user its paw for a handshake.

Hardware communication with the robot is not as easy as in previous Pleo commercial versions. In order to obtain data in “real time” Pleo was connected to an external power source while performing communications between its USB connector and a remote computer. It is possible to get values from Pleo’s sensors in real time using this system setup. It is also possible to connect via bluetooth to a UART port [12] to bridge Pleo and a Raspberry Pi. Other processing platforms like Intel Galileo or Edison, and wireless communications like ZigBee or WiFi can also be implemented depending on power, processing, and privacy needs. The Raspberry Pi module can access the Internet via a Wi-Fi dongle to upload data to the cloud.

There were two problems in this communication setup: firstly there was a lack of physical space to work with since the original battery of Pleo was employed with compacting wires and left the bluetooth module visible. Secondly, new Pleo robots are not equipped with this UART connector, so they could be exclusively tested in old versions.

### Cloud-based Pleo robot

**Fig. 1:**
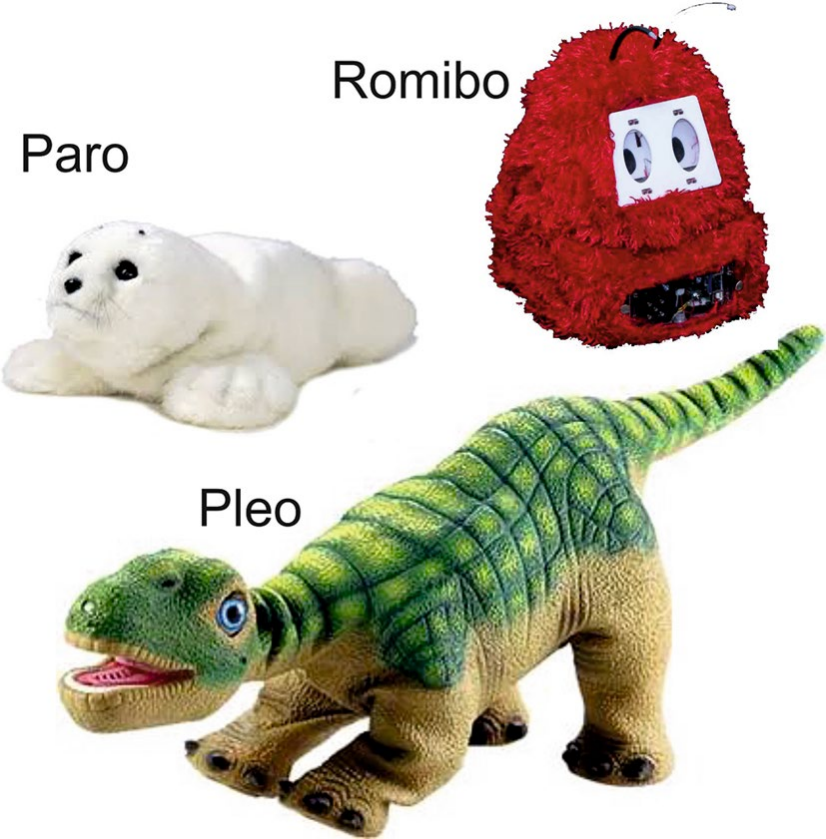
Therapeutic robots. Commercial therapeutic robots alternatives

In order to expand Pleo’s connectivity it was transformed into a cloud client that minimized the impact on the hardware that already existed. The implementation is done by not changing the pre-programmed bio-inspired (Fig. 1) behaviour, doing zero modifications on the embodiment, and keeping a good trade-off between data transfer and power consumption between the robot and the cloud.

**Fig. 2:**
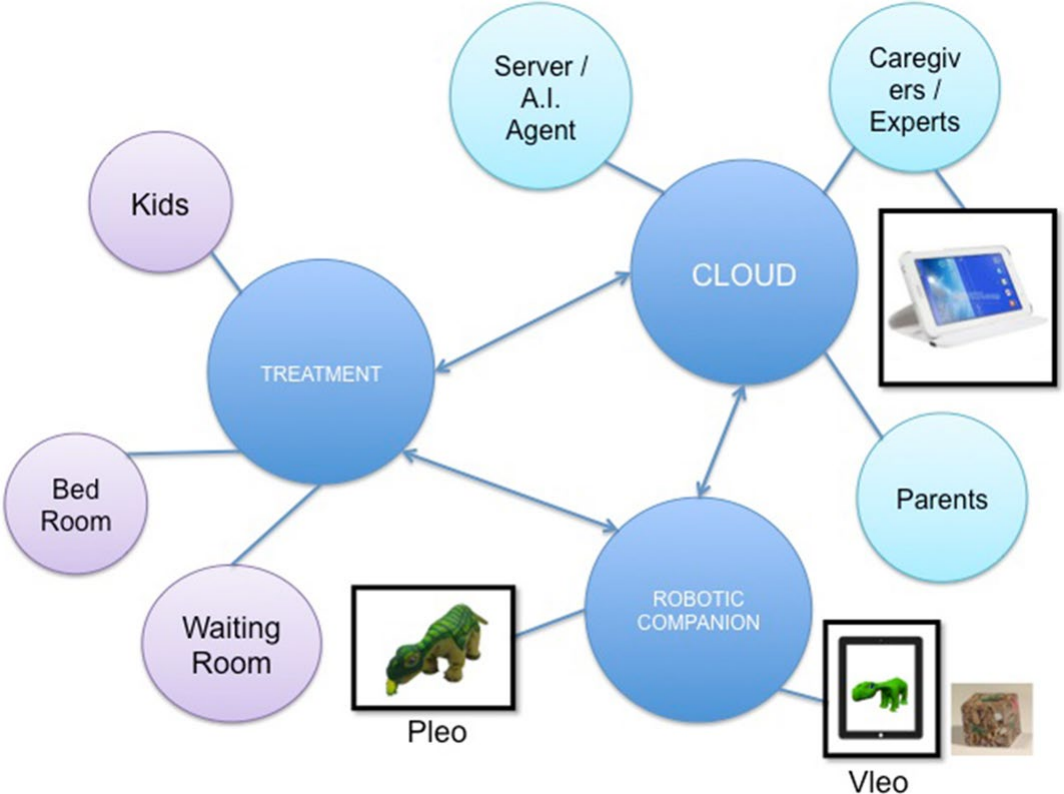
Cloud of Pleo robots. Conceptual overview of the cloud of pet robots

Figure 2 shows a cloud architecture that allows human users and artificial intelligent agents to stimulate the pet robot by modifying its internal states. It depicts a conceptual overview of the overall project where the three key elements are the cloud, the robot companion, and the therapy done by the Pleo robot.

The three stimulation elements in this cloud robotics design that can modify Pleo’s internal states through the cloud are [13]:

- *The artificial intelligent agent*—Is aimed at learning which decision parameters and descriptors are relevant. This element is currently under development, so it is out of the scope of this work.
- *The interface*—Induces customized behaviour to the Pleo by changing its parameters. This element has been currently developed as an Android app along with physical modifications for wireless communication.
- *The VLEO, a tangible user interface*—Combines a virtual avatar with augmented reality, to reproduce the Pleo robot. It interacts with other pet robots on the cloud. This element is under development and early results are introduced in this text.

### VLEO: the virtual social character that interacts with Pleo

VLEO is a smart avatar. What makes VLEO unique is that it is a virtual character that interacts with both humans and Pleo robots. It is proven that tangible devices increase the quality of interactions compared to those interactions with virtual agents. A down side is that physical agents are constrained by their size and shape as well as their environment. By combining a virtual agent and a physical robot it is possible to increase the complexity of an interaction through narratives and virtual worlds. Our early tests involve children from 8 to 12 years old (11 boys and 4 girls) [13]. Observations suggested that virtual social robots could improve engagement and enjoyment during a child’s play time. 83.3 % preferred to play with VLEO and the Pleo and 16.7 % preferred only Pleo.

VLEO’s controller is a cube. Each face of the cube represents a Pleo’s state change. By turning the cube children can modify the states of Pleo. The cube’s faces have a pattern that is read by the camera of a tablet, and the tablet is connected to the cloud server where the game engine (Unity) enables a graphic rendering of a virtual Pleo. The visual tracking of the cube is done by using Vuforia Unity Extension, and the magnitude of the state change is controlled by the angle of the cube. Pleo’s connectivity to the server is done through a local processor (computer or Raspberry Pi) using the battery system with bluetooth.

### Externalizing Pleo’s moods and states through bluetooth

Unfortunately, earlier workshops run by a psychologist and a pediatric group with children from the HSJD hospital have been performed without communication systems. When the Pleo had a bad mood or acted in a non-normal way there was no manner to know exactly what was happening. Pediatric groups could only correct the situation based on their personal knowledge.

The objectives pursued in this work (an extension of the work developed in [14]) are presented as the following:

- Provide an extension for Pleo that can be mounted in any Pleo for bluetooth communication.
- Give the coordinator the ability to control Pleo in order to make a specific action due to a situation. An Android application was chosen to run this platform since its open source software makes it convenient to use and easy to make changes.
- Prove a bluetooth-battery package that fits inside the battery hole in order to assemble the module in one package.

## Results

### Hardware

**Fig. 3:**
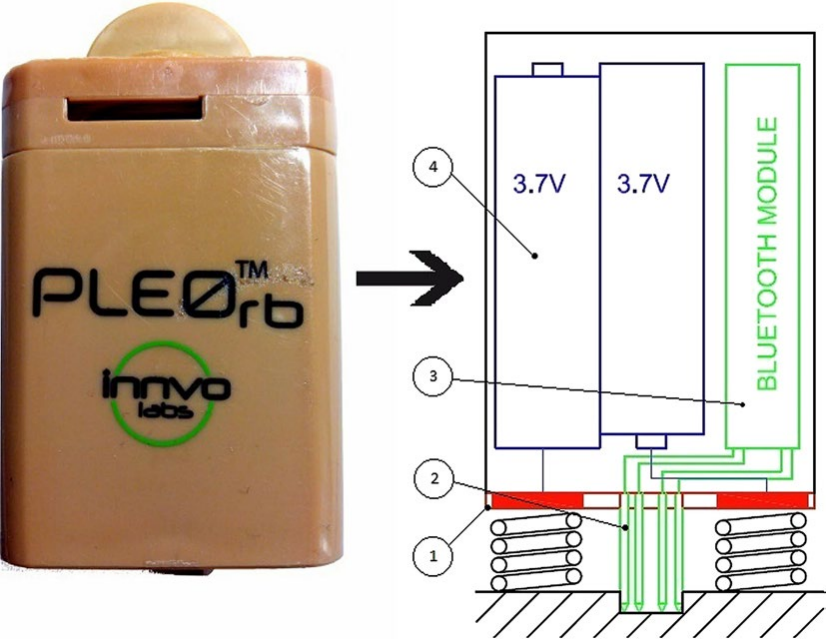
Layout of the proposed assembly. Battery pack with bluetooth embedded

The proposed solution for the hardware challenge, shown in Fig. 3, is to switch Pleo’s battery for a battery-bluetooth package. Distinguishable components are listed below.

- PCB: Its main function is to act as the conductive element between the batteries and the springs that feed the robot. Based on the base of the battery, it must fix the 4 pins that establish contact to the bluetooth output of Pleo.
- Connector pogo pins: Catch the signal that Pleo sends to be processed in the bluetooth module.
- Bluetooth module: Receives the data signal from the robot and sends it to the connected device. The JY-MCU and HC-05 are two of the cheapest Bluetooth serial port modules in the market, but their provided voltage is not enough. A more sophisticated module is needed, such as the RN-41 microchip.
- Battery pack: Pleos battery requites a 7.4 V, a charge of 2800 mAh, 20.72 Wh power and can withstand a max temperature of 60 °C.

A 3D printed case was made for the elements of this assembly.

### Interface

**Fig. 4:**
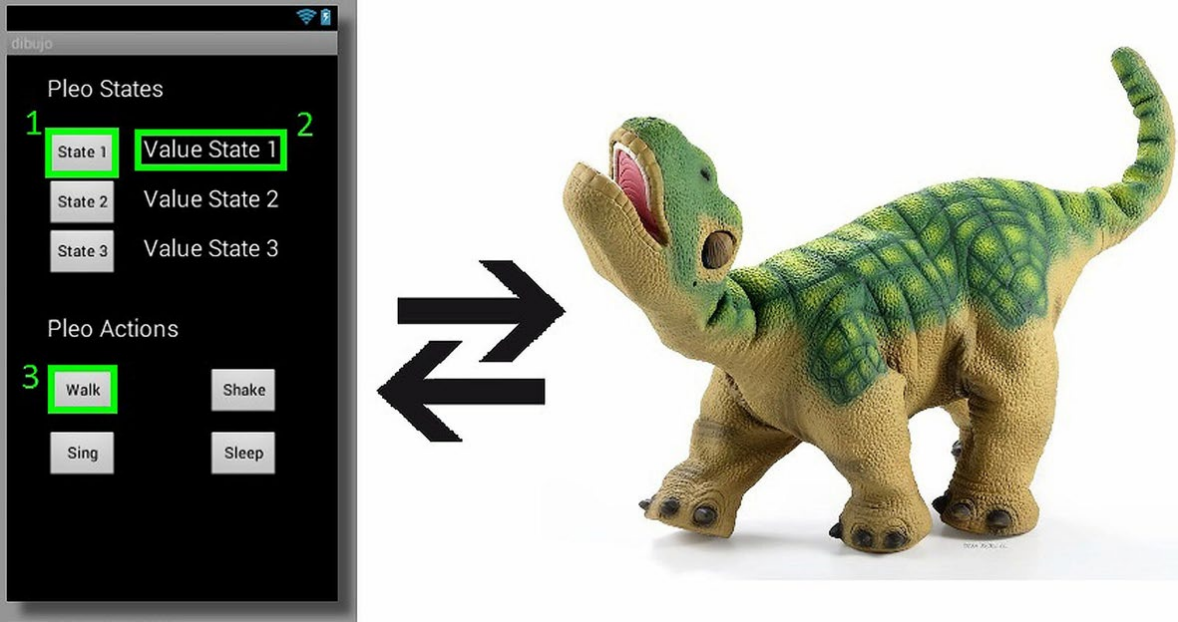
First sketch of the interface. First sketch of the Android application

The interface must be as simple and comfortable for the user as possible. Connecting to the Pleo robot must be user friendly, bluetooth must turn ‘on’ when the app is launched and a button should be present to search Pleo’s signal and establish communication. A first sketch of the main menu is shown in Fig. 4. It presents a list of different Pleo states, allows the user to modify the emotional status of Pleo, and allows the user to ask for their value. If needed, it is possible to add more buttons for Pleo actions, in order to respond with a specific action when the situation requires it.

### Development of the solution

#### Connection via terminal

Pleo USB connection does not support Win 64 bits, so a virtual image for XP 32 bits was implemented. The easiest way to connect to the Pleo is via PuTTY and USB connection.

#### Bluetooth assembly

One of hardest challenges in this work is the challenge involved in building a Bluetooth assembly. On the rear face of the battery inside the hole it is impossible to insert a vernier caliper for measuring purpose. Moreover the rear is a little wider than the external perimeter of the hole.

To solve the problem of obtaining the correct pin positions, a thin sheet of transparent plastic was cut, introduced inside the hole and marked with a fine-tipped pen. The plastic was then extracted, measured with the vernier caliper and drawn with a CAD tool. Once the layout was drawn, the next step was to build the PCB. Finally, a 10 × 1 female header was welded to connect the RN-41 without welding directly to the module that could damage it.

#### Power issues

When the PCB is set inside the Pleo, batteries power the robot, but after pressing its button to turn it on, the robot does not move. It was believed that both batteries were not enough to power the robot and the Bluetooth module, so a button cell of 3.3 V was used to power the RN-41, without taking up too much space. After these changes Pleo’s awoke, sent some data to the screen of the terminal, but soon afterwards it powered back off; canceling communication.

It seemed like a power failure, hence the minimum tension and intensity to make the robot work was measured. There it was discovered that the minimum power to supply to Pleo RB is 7.4 V (equivalent to 3A). Placing the robot in sleep mode was done to reduce its power values a little bit. This explains why the same battery that Pleo carries provides more than 8.4 V when it is completely full. Another discovery was that the Pleo robot, unlike USB wired communication, does not allow bluetooth communication if the robot is not running.

#### Android app

**Fig. 5:**
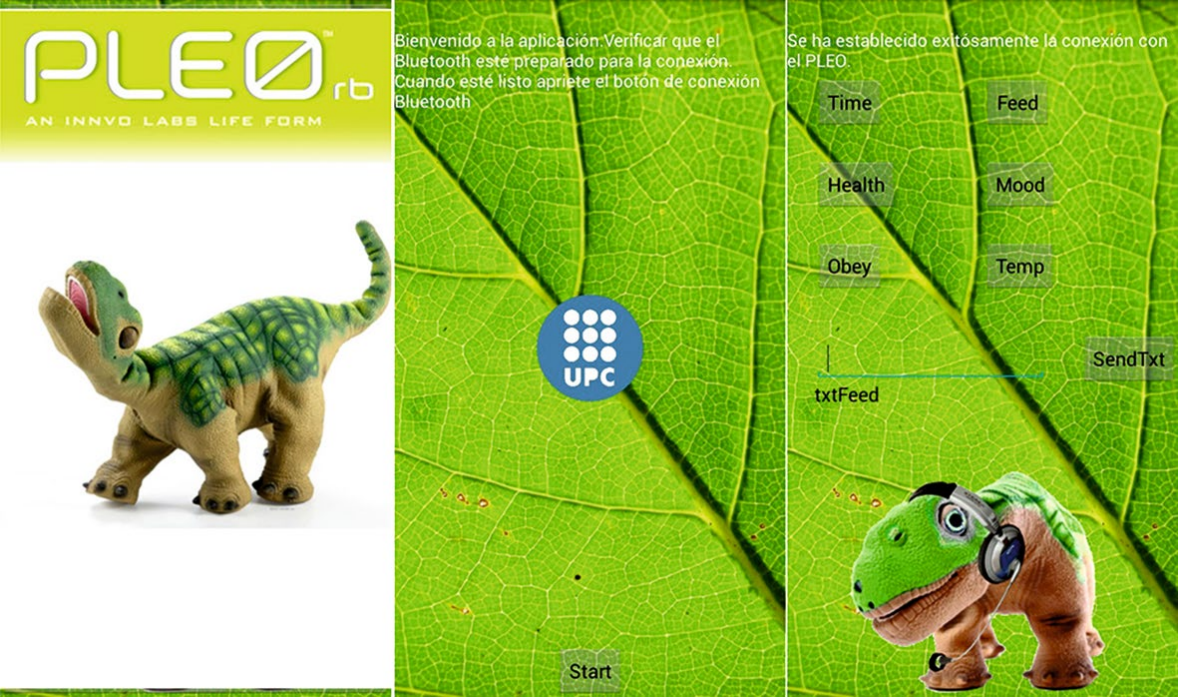
Android application interface. Final Android application

Figure 5 shows how the interface looks like. The first scene is a presentation to introduce the user. Here the application turns ‘on’ the Bluetooth of the smartphone or device where the app is installed. In the next scene there is a button that user must push once the module RN-41 is powered in order to pair it with the device. Then the app searches the corresponding MAC direction. In the case that it does not find it, it will display an error message. On the other hand, if the communication is established the app redirects the user to the third scene (command window), where the user sends and receives data from Pleo. When Pleo is switched on, unlike USB communication, the robot starts sending data on the initialization of the source. Hence, the application developed cannot receive Pleo’s states values or send any motion command until initial conditions has been received and processed.

## Conclusions

Wireless communication between Pleo and an Android device is achieved. With the Android app developed the user is able to obtain any state of the robot without stopping its interaction with the patient. It also contains a revisable text box to allow the user to send orders to the robot from a list of allowed actions.

Communication with the robot with the bluetooth module to the output has been successful. As newest Pleo’s versions have no UART port connection, upcoming works with Pleo may now look to a solution based on what has been explained in this work. Bottom connections are insured to work properly and can be used in further research.

During test trials, it has been made evident that using a couple of 3.7 V cells is not enough to make Pleo run due to these values being too close to the operating limit. Using a third cell is needed to make the Pleo robot move.

Moreover, the developed Android app can be installed in many devices and its interface is easily understandable for users.

After many hours working with Pleo, several observations can be expressed about it, which three have been highlighted and described below:

- Pleo is not a robust platform. It is common with Pleo that it may during some days not turn on. If Pleo survives for more than 1 month without these boot up issues it should be considered economical since it is affordable.
- Pleo is unable to react to a child’s laugh or cry. This is a very important issue because it leads to a child losing interest, and is known that the progress of the therapy is directly proportional to the motivation of the patient. If Pleo does not seem to care for the child, the child will not care of Pleo and the therapy could fail.
- In the future it is desired for the cloud to be able to collect the states of these children.

One of the first milestones of this project, the Bluetooth communication with Pleo, has been achieved completely. Using the PCB with the extensible pins it is possible supply. Bluetooth communication to any Pleo, even those without UART connection. The second milestone, which is to modify Pleo’s states from an Android device, has also been successfully achieved. This has helped the coordinator understand and control the robot through a user friendly app. The third milestone, which was to assemble a bluetooth and battery package has not been fully completed. Pleo works using 3 AA cells and fits to the designed package in this work. This setup was not completely developed, since a 3D cage was built for two AA cells. It should be easy to build a newer more compatible assembly.

Pleo is a commercial closed platform, thus alternative platforms should be considered in the near future. In this sense, two technical research lines are opened. The first one involves consideration to commercial platforms and design of a pilot robotic platform to be certified for commercial use. The second involves working with the possibility to certify the employed device as a medical device for therapeutic purposes.
